# What makes the dorsomedial frontal cortex active during reading the mental states of others?

**DOI:** 10.3389/fnins.2013.00232

**Published:** 2013-12-05

**Authors:** Masaki Isoda, Atsushi Noritake

**Affiliations:** Department of Physiology, Kansai Medical University School of MedicineHirakata, Japan

**Keywords:** dorsomedial frontal cortex, theory of mind, mentalizing, self, others, executive function, intention, uncertainty

## Abstract

The dorsomedial frontal part of the cerebral cortex is consistently activated when people read the mental states of others, such as their beliefs, desires, and intentions, the ability known as having a theory of mind (ToM) or mentalizing. This ubiquitous finding has led many researchers to conclude that the dorsomedial frontal cortex (DMFC) constitutes a core component in mentalizing networks. Despite this, it remains unclear why the DMFC becomes active during ToM tasks. We argue that key psychological and behavioral aspects in mentalizing are closely associated with DMFC functions. These include executive inhibition, distinction between self and others, prediction under uncertainty, and perception of intentions, all of which are important for predicting others' intention and behavior. We review the literature supporting this claim, ranging in fields from developmental psychology to human neuroimaging and macaque electrophysiology. Because perceiving intentions in others' actions initiates mentalizing and forms the basis of virtually all types of social interaction, the fundamental issue in social neuroscience is to determine the aspects of physical entities that make an observer perceive that they are intentional beings and to clarify the neurobiological underpinnings of the perception of intentionality in others' actions.

## Introduction

The success of human life depends on interactions with other individuals. The social world thus constantly prompts one to reflect upon both one's own mental states (e.g., thoughts, intentions, desires, and beliefs) and those of others. The ability to explain and predict others' behavior in terms of their mental states is known as having a theory of mind (ToM) or mentalizing (Baron-Cohen et al., 1985, 1999; Frith and Frith, 1999). This ToM ability is the basis for many social behaviors such as cooperation, reciprocity, empathy, and deception. Studies using functional magnetic resonance imaging (fMRI) have consistently demonstrated that the dorsomedial frontal cortex (DMFC) is a core component in mentalizing networks (Gallagher and Frith, 2003; Amodio and Frith, 2006). In such studies, the foci of DMFC activation can range from Brodmann area 6 (BA 6) (Baron-Cohen et al., 1999), which may roughly correspond to the pre-supplementary motor area (pre-SMA), to BAs 8 and 9 (Fletcher et al., 1995; Goel et al., 1995; Happe et al., 1996; Gallagher et al., 2000) and further anteriorly to BA 10 (Amodio and Frith, 2006; Gilbert et al., 2006). Anatomical connections between the pre-SMA and anteriorly adjacent areas of the frontomedian wall (Luppino et al., 1993; Johansen-Berg et al., 2004; Yeterian et al., 2012) suggest their functional integrity. In parallel with fMRI findings, clinical case studies have also shown that patients with DMFC lesions can exhibit severe ToM impairments (Happe et al., 1999; Rowe et al., 2001; Stuss et al., 2001). These findings collectively implicate the DMFC in ToM.

Then, why is the DMFC generally activated during ToM tasks at all? What component processes of ToM, if any, are responsible for activating the DMFC? There has been a debate regarding domain specificity vs. domain generality of ToM. One view posits that ToM depends on functional modules that are specialized for ToM computations (domain specificity) (Leslie and Thaiss, 1992; Baron-Cohen et al., 1999; Frith and Frith, 2003; Saxe et al., 2004). The other view claims that ToM can be accounted for by the integration of multiple functional modules, each of which is not originally specialized for social cognition (domain generality) (Carlson et al., 2004; Apperly et al., 2005; Stone and Gerrans, 2006). One confounding factor that might make this issue controversial is the inclusion of any material in cognitive tasks that, by itself, activates mentalizing processes (Van Overwalle, 2011). Indeed, even abstract shapes that move in a biologically plausible manner, verbal stories or cartoons that involve goal-directed actions, or traits that are suggestive of social beings can all automatically recruit the ToM network (Van Overwalle and Baetens, 2009). However, our goal is not in the in-depth discussion on such an intractable debate; the issue is beyond the scope of this study. Instead, the goal of this article is to address potential relationships between the DMFC and several processes that may be closely associated with ToM. In particular, we will illuminate executive inhibition, self-other distinction, prediction under uncertainty, and perception of intentions, and discuss how the DMFC participates in each of these processes. What the four processes have in common is twofold. First, they are all associated with the process of predicting others' intention, a crucial aspect of ToM for understanding and anticipating others' behavior (see below). Second, it is becoming technically feasible to investigate their cellular mechanisms using the single-neuron recording method in non-human primate platforms. Thus, our intention is to incorporate recent progress on the cellular basis for predicting others' intention into the dominant literature in developmental psychology and human neuroimaging. We believe that the functional imaging technique and single-neuron recording technique will complement each other to uncover the cellular and network mechanisms of ToM. Note that our position does not immediately support domain generality of ToM. As will be discussed later, viewing a physical entity as an intentional being might be a mental process that is uniquely social. This mental process may be deeply related to an indeterministic bias or moral responsibility that people typically attribute to social agents, but not to non-social objects (Nichols, 2011).

In what follows, we review the experimental findings from different disciplines, in particular, developmental psychology, clinical neuropsychology, human neuroimaging, and electrophysiological recording in monkeys. Although monkeys may not mentalize as humans do, they possess related skills. Monkeys can actively monitor a conspecific's actions and their outcomes for planning their own actions (Yoshida et al., 2011, 2012; Chang et al., 2013). They can make inferences about what others can see (Flombaum and Santos, 2005). Supporting this view, the DMFC of humans and monkeys, including areas associated with ToM, has functional organization that shares similar patterns of coupling between each DMFC subregion and the rest of the brain (Sallet et al., 2013). There has been no evidence for “new” regions in the human DMFC (Sallet et al., 2013). Moreover, the increased complexity of monkeys' social environments is accompanied by an increase in the volume of the gray matter in the DMFC (Sallet et al., 2011). These findings suggest that the DMFC plays an important role in social cognition in monkeys as well.

## Executive inhibition

The construct of executive functions subsumes several processes that allow for generating flexible thought and behavior. Executive control includes inhibition, shifting, updating, access, working memory, and planning (Miyake et al., 2000; Fisk and Sharp, 2004; Baez et al., 2012) and can effectively integrate cognition and emotion (Pessoa, 2008), so that organisms can guide an appropriate decision in novel or dangerous situations while suppressing a pre-potent, habitual action that is no longer appropriate (Shallice, 1998). Among several executive processes that are potentially associated with ToM (Aboulafia-Brakha et al., 2011; but see Baez et al., 2012 for an alternative view in people with autism spectrum disorders, ASDs), executive inhibition–i.e., deliberate suppression of immediate behavior in order to achieve a later, internally represented goal (Nigg, 2000)—has been most consistently reported to be a crucial factor enabling the development of social competence such as ToM (Carlson and Moses, 2001; Carlson et al., 2004) and cooperation (Ciairano et al., 2007). In support of this view, executive inhibition is impaired in children with ASDs (Ozonoff et al., 1991; Frith, 1997; Robinson et al., 2009), whose performance of ToM tasks is severely impaired (Baron-Cohen et al., 1985).

The close association between executive inhibition and social cognition, in particular ToM, is rooted in the saliency of self-relevant information as well as people's habitual tendency to use themselves as the reference point in social judgments, which is sometimes referred to as the “egocentric assumption of shared perspectives” (Fenigstein and Abrams, 1993) or “epistemic egocentrism” (Royzman et al., 2003). For example, recall of self-relevant information is better than recall of other kinds of information (Rogers et al., 1977; Bower and Gilligan, 1979). Self-relevant information enjoys privileged accessibility, greater confidence, and reduced response time compared with other-relevant information (Rogers et al., 1977; Bower and Gilligan, 1979; Kuiper and Rogers, 1979; Aron et al., 1991). Furthermore, people tend to impute pre-potent self-perspective to others (Moore et al., 1995; Mitchell et al., 1996; Nickerson, 1999). These biases, however, can give rise to a potential problem of correctly attributing a mental state to its proper agent, leading to misapprehensions of others' minds. These psychological observations have led Decety and Sommerville (2003) to argue that executive inhibition may be a necessary requisite to suppressing the pre-potent self-perspective in favor of others' discrepant perspective when reading the mental state of others. Consistent with this view, children with poor executive inhibition have problems in social relationships owing to the poor ability to recognize others' desires (Henker and Whalen, 1999). In older adults as well, the reduced ability to inhibit pre-potent self-perspective is associated with the difficulty in taking the perspective of another (Bailey and Henry, 2008). Of interest is that a patient with damage in the right inferior frontal gyrus (rIFG) is able to infer another's state of mind when he himself does not hold a strongly conflicting self-perspective (i.e., low self-perspective inhibition demands); however, the patient performs poorly in tasks with high self-perspective inhibition demands (Samson et al., 2005). The rIFG has long been thought to play a role in executive inhibition in non-social contexts (Konishi et al., 1998; Aron et al., 2004; Chambers et al., 2006). Yet, evidence is now accumulating to support the existence of shared neural substrates for inhibitory control in complex social situations and basic motor response inhibition (Brass et al., 2005; Samson et al., 2005; van der Meer et al., 2011).

**Figure 1:**
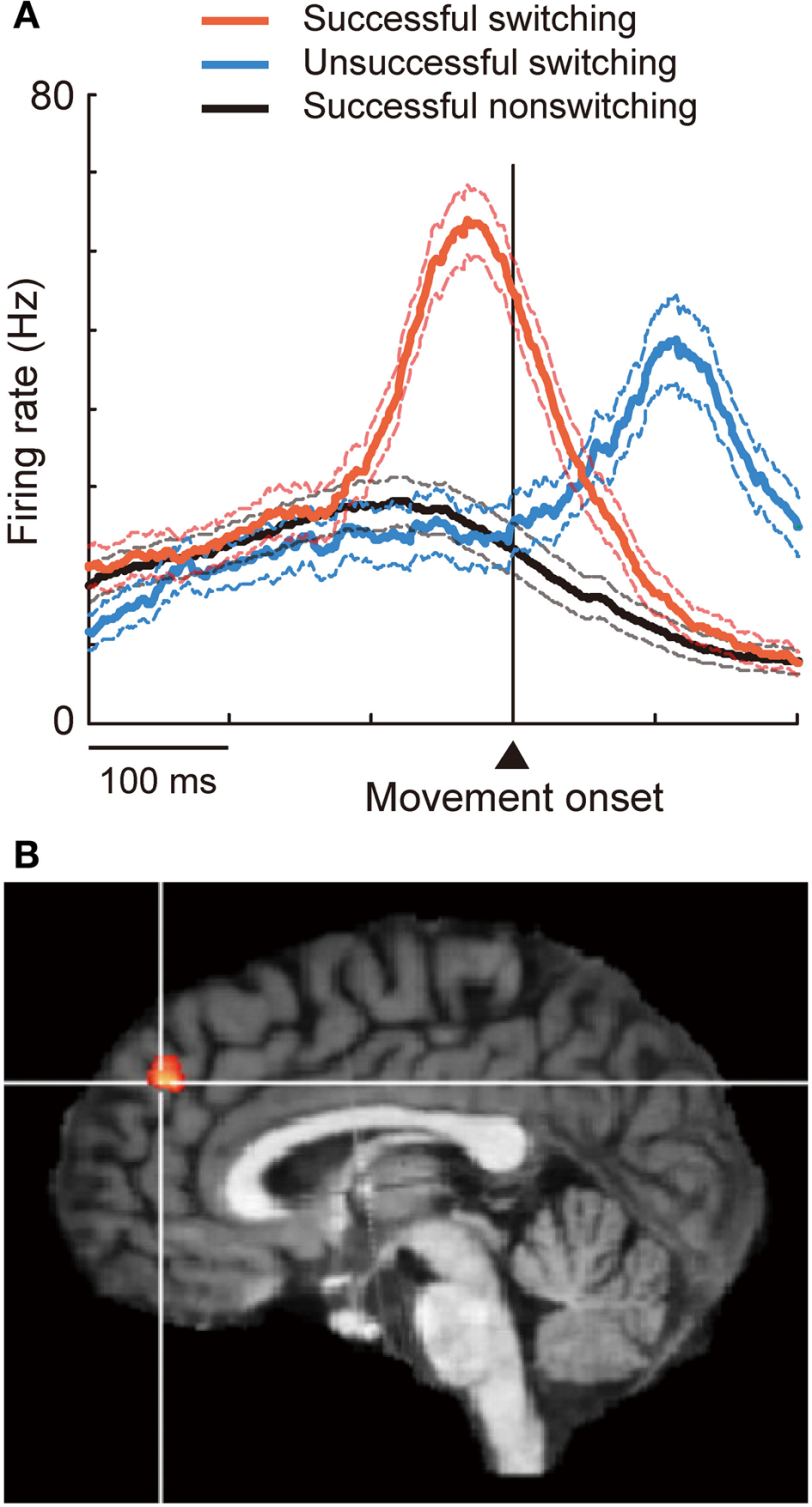
**Involvement of DMFC in executive inhibition. (A)** When the animal successfully switched to a now-valid action by inhibiting a pre-potent, no-longer-valid action, pre-SMA “switch neurons” fired phasically before movement onset (red). Switching was unsuccessful when the initiation of activity increase was delayed (blue). **(B)** The contrast between inhibition vs. action trials revealed activation in the dorsal frontomedial cortex (BA 9). Reprinted with permission from Brass and Haggard (2007).

The DMFC constitutes another critical node subserving inhibitory control. This was first demonstrated by Penfield and Welch (1949) more than 60 years ago. They noted that electrical stimulation in the human DMFC suppressed voluntary movement, typically characterized by slowing, hesitation, or inability to initiate or continue phasic motor activity without affecting consciousness. Since then such “negative” motor phenomena have been consistently reported as the inhibitory effects of stimulation on motor performance (Lim et al., 1994; Luders et al., 1995; Yazawa et al., 2000; Yamamoto et al., 2004) and as readiness potentials preceding voluntary muscle relaxation (Terada et al., 1995; Yazawa et al., 1998). Recently, the role of the DMFC in executive inhibition has been characterized using more demanding behavioral tasks. For example, the DMFC, particularly the pre-SMA and nearby regions (Figure 1A), is activated when subjects suppress an impending action or a cognitive set particularly under the presence of strong response interference or in favor of alternative, less-dominant options (Ullsperger and von Cramon, 2001; Garavan et al., 2003; Nachev et al., 2005; Aron et al., 2007; Isoda and Hikosaka, 2007; Duann et al., 2009; Hikosaka and Isoda, 2010; Konishi et al., 2010; Sharp et al., 2010; Duque et al., 2013);. Electrical stimulation in the DMFC can inhibit the generation of eye movement, but this effect is only observed when the stimulation is delivered after a cue is given to initiate the movement (Isoda, 2005). Executive inhibition can be impaired in subjects with superior DMFC damage (Floden and Stuss, 2006) or in intact subjects with stimulation (Chen et al., 2009; Hsu et al., 2011) applied over the same DMFC region. The inhibitory control of the DMFC may be mediated by interaction with other cortical regions such as the rIFG and primary motor cortex, and/or with subcortical regions such as the subthalamic nucleus (Johansen-Berg et al., 2004; Aron et al., 2007; Taylor et al., 2007a; Isoda and Hikosaka, 2008, 2011; Duann et al., 2009; Mars et al., 2009; Neubert et al., 2010; Duque et al., 2013).

Executive inhibition has been typically mapped in the pre-SMA, the rostralmost part of BA 6 within the DMFC (Van Overwalle, 2011). Other neuroimaging studies, however, point to the involvement of more rostral regions as well. Most of the studies outlined above have focused on inhibitory control elicited by external stimuli. However, in daily life people very often decide themselves whether to or not to act. Incorporating this critical aspect of inhibition in a task paradigm has revealed that the dorsal frontomedian cortex (BA 9; Figure 1B) is involved in “self-control” of inhibition (Brass and Haggard, 2007; Kuhn et al., 2009). A similar brain region is also activated when participants themselves decide to quit continued gambling to recover previous losses (loss chasing) (Campbell-Meiklejohn et al., 2008). Furthermore, even more rostral regions (the anterior frontomedian cortex, BA 10/32) come into play when people inhibit automatic tendencies to imitate others (Brass et al., 2005, 2009). Many motor skills, language, and moral behaviors are learned via imitation in earlier life, but adults do not generally imitate others very often. In fact, people might become irritated when someone else intentionally imitates them. In this light, imitation inhibition is socially adaptive. These findings suggest that the DMFC plays a key role in executive inhibition, with more rostral regions being increasingly recruited as the degree of self-control or a social need increases. Future studies should explicitly address the question of whether the DMFC also plays a role in inhibiting pre-potent self-perspectives.

## Distinction between self and other

There is converging evidence from different disciplines that the perception and execution of an action have a common representational basis. First, it has been documented in cognitive psychology that the observation of an action automatically primes a corresponding motor representation in the observer. For example, the execution of an action (e.g., index finger movement) while observing an incongruent action (e.g., middle finger movement) leads to a longer reaction time than while observing a congruent action (Brass et al., 2009). Intriguingly, observed environmental constraints are also automatically mapped onto the observer's motor system: observing another's hands being physically restrained leads to a longer response time (Liepelt et al., 2009). Second, evidence from clinical neuropsychology shows that people with frontal damage can display echopractic responses. For example, when patients are instructed to show their index finger upon seeing the experimenter's fist but to show their fist upon seeing the experimenter's index finger, they tend to copy the observed action (Luria, 1980). Moreover, prefrontal patients can show strong imitative response tendencies even when not instructed to do so (Lhermitte et al., 1986). Finally, evidence from neuroscience clearly demonstrates that common coding occurs between perception and action at the level of single neurons in various parts of the brain (Rizzolatti et al., 2001). These neurons, called “mirror neurons” and originally found in the monkey brain, are hypothesized to play a role in understanding others' actions and goals (Rizzolatti et al., 2001). Taken together, these findings support the existence of mirror-matching mechanisms in the central nervous system, whereby perceiving an action automatically activates the equivalent motor representation in the observer.

However, people do not normally confuse others with themselves. This is true even when the other is produced by the imagination of the self. People are readily capable of attributing actions to either themselves or another. The classical mirror-matching theories are silent on how the brain carries out such attribution. Despite ample evidence for the shared self-other representation, there must exist a mechanism that separates self- and other-related motor representations (Jeannerod, 1999). A previous study supports the idea that the motor system represents other agents as qualitatively different from the self (Schutz-Bosbach et al., 2006).

The formation of mentalizing capacity necessitates the ability to form the representation of others' mental states and to distinguish it from one's own (Frith and Frith, 1999). As mentioned earlier, we tend to view others as analogous to ourselves, but we also identify them as unique. In the social world, we reflect not only upon our own mental states, but those of others around us as well. Moreover, such mental states must be correctly assigned to their proper agent. This capacity may prevent self-other confusion and chaotic social interactions, as is the case in people with schizophrenia who demonstrate overextension of agency to others' actions or attenuation of self-agency (Decety and Grezes, 2006). In the laboratory, mentalizing capacity is evaluated most often using false belief tasks that require distinction between one's own and others' beliefs. Children with ASDs show a marked difficulty dissociating a false belief of another person from their own true belief. It has been argued that individuals with ASDs are strongly self-focused, which is hypothesized to arise from the lack of distinguishing between self and another (Lee and Hobson, 2006; Mitchell and O'Keefe, 2008; Lombardo et al., 2010a). The self-other distinction is also central to self-consciousness and agency (Decety and Grezes, 2006).

The ability to distinguish between self and others appears to develop throughout the infancy period (Sebastian et al., 2008; Burnett and Husain, 2011). For example, newborn babies orient their face toward the source of tactile stimulation more frequently to external touch than to spontaneous self-touch to the cheek (Hespos and Rochat, 1997). By 5–6 months of age, infants preferentially view a video of another infant compared with a video of themselves (Bahrick et al., 1996). Children start to recognize themselves in mirrors at around 18 months (Povinelli, 1995). In the second and third years, infants start to understand that others are similarly self-aware and differentiate between themselves and another in speech (Bates, 1990). These empirical observations are considered to be evidence for having neural mechanisms that distinguish between self and others.

Accumulating evidence indicates that, unlike the mirror system, self- and other-related processes can be segregated in the DMFC. Neuroimaging studies have shown that self-related judgments are associated with the ventral MFC (BAs 10 and 32), whereas other-related judgments are associated with the DMFC (BAs 8 and 9) (Van Overwalle, 2009; Denny et al., 2012). Crucially, the z-coordinates in individual studies can predict whether the study involves self- or other-related judgments, which are associated with increasingly ventral or dorsal portions of the MFC, respectively (Denny et al., 2012). Such an areal segregation appears to depend on the perceived overlap between self and others (in terms of sociopolitical views), as mentalizing about a *similar* other engages a region of the ventral MFC that is linked to self-referential thoughts, whereas mentalizing about a *dissimilar* other engages a more dorsal region of the MFC (Mitchell et al., 2006). It should also be noted, however, that Behrens and co-workers propose another view that a functional gradient in the MFC is better tied to the relevance of valuation for current choice (executed values vs. modeled values) than to the frame of reference of the individual (self vs. other) (Nicolle et al., 2012). In addition to the ventral MFC, neurotypical individuals preferentially recruit the middle cingulate cortex during self-related processing compared with other-related processing (Mitchell et al., 2006; Tomlin et al., 2006; Chiu et al., 2008; Lombardo et al., 2010a). However, individuals with ASDs display the reverse or lack of the preferential response to the self in the middle cingulate cortex (Chiu et al., 2008; Lombardo et al., 2010a) as well as the ventral MFC (Lombardo et al., 2010a). This atypical neural self-other distinction may mirror atypical behavioral self-other distinction in ASDs (Lee and Hobson, 2006; Mitchell and O'Keefe, 2008; Lombardo et al., 2010a).

**Figure 2:**
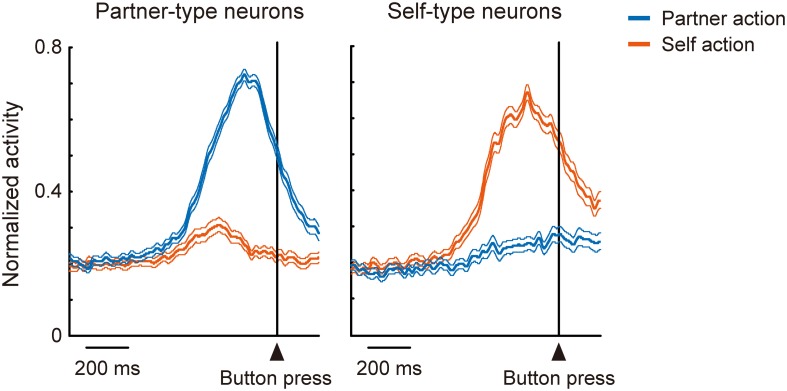
**Involvement of DMFC in self-other distinction**. A group of DMFC neurons (“partner-type neurons”) were preferentially activated when the recorded monkey observed another monkey making an action (blue), while another group of DMFC neurons (“self-type neurons”) were preferentially activated when the recorded monkey executed an action (red).

In the mirror system, coding of one's own actions and others' actions overlaps at the level of single neurons. How then do individual neurons in the mentalizing system, in particular the DMFC, code the two kinds of action? The ability to mentalize might have evolved from a system for representing actions (Frith and Frith, 1999), as action is one of the main channels used for interpersonal communication. Determining the agent of action may thus contribute to the differentiation of self and others (Jeannerod, 1999). To address this issue, Isoda and coworkers trained two monkeys sitting face-to-face to perform a role-reversal task (Yoshida et al., 2011, 2012). In each trial, one monkey was assigned the role of an actor and the other an observer, and the roles alternated every two trials. During each trial, the actor made a choice between a yellow or green illuminated button. If the actor made the correct choice, both monkeys received a reward. Thus, reward expectation was constant across two animals in each trial, and the experimenters were able to identify agent-specific neuronal signals. They found that “partner-type neurons”—which fired selectively during the partner's action (Figure 2, *left*)—were encountered significantly more frequently in the pre-SMA and its anterior extension including BA 8 possibly extending into the caudal BA 9, whereas “self-type neurons”—which fired selectively during one's own action (Figure 2, *right*) —were significantly more prevalent in more ventral, cingulate sulcus regions including the rostral cingulate motor area and its anterior extension (Yoshida et al., 2011). These findings support the hypothesis that self-actions and others' actions are differentially represented in the DMFC. The findings are also consistent with human fMRI findings showing that attribution of other-agency activates the pre-SMA and BA 8 (Sperduti et al., 2011). An important issue to clarify in the future is the computational operation whereby distinction between aspects of self and others is accomplished (Blakemore et al., 2002). Very recently, a coordinate transformation approach has been proposed to account for such operations (Chang, 2013; Chang et al., 2013).

## Prediction under uncertainty

The mental states of others are much less predictable than those of one's self. This may be particularly true for distant others as opposed to close others, and under competition as opposed to cooperation. Unpredictability of others' minds may be rooted in asymmetry of information sources that people use to make inferences about self and others. Specifically, the information people use for themselves is largely introspective and interoceptive, whereas the information available to infer about others is largely extrospective and exteroceptive (Lombardo and Baron-Cohen, 2011). That is, one cannot directly access the sensation, emotion, or thought of others. Instead, one's experience of others' phenomenology is primarily dominated by observing their external behaviors (Pronin, 2008). Reading others' minds is thus inherently an uncertain process. It is therefore possible that brain regions processing uncertainty come into play during mentalizing about others.

From a deterministic viewpoint, uncertainty is always caused by a lack of knowledge. Nevertheless, uncertainty has been operationally divided into two constructs: risk or expected uncertainty, and ambiguity or estimation uncertainty (Knight, 1921; Payzan-LeNestour and Bossaerts, 2011; O'Reilly, 2013). Risk or expected uncertainty refers to the type of uncertainty that derives from stochasticity inherent in the environment, where variance determines the level of uncertainty. This type of uncertainty is what we cannot control and is therefore attributed to external reasons (Howell, 1971; Kahneman and Tversky, 1982). In contrast, uncertainty that arises from people's insufficient knowledge is referred to as ambiguity or estimation uncertainty. This type of uncertainty is attributed to internal factors and can be reduced by obtaining more pieces of information. It seems likely that uncertainty associated with inferring others' mental states or predicting others' behavior does not originate from stochasticity of the world around us, but is due mostly to internal factors, that is, ambiguity or estimation uncertainty. Thus, better understanding of others requires constantly updating the current belief about them on the basis of incoming information obtained through observation (Behrens et al., 2008).

**Figure 3:**
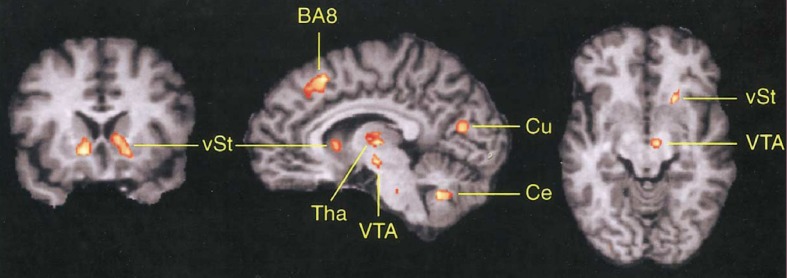
**Involvement of DMFC in prediction under uncertainty**. The contrast between prediction under uncertainty vs. control conditions revealed activation in several brain regions including the frontomedian cortex (BA 8). vST, ventral striatum; Tha, thalamus; VTA, midbrain area; Cu, cuneus; Ce, cerebellum. Reprinted with permission from Volz et al. (2003).

As can be seen in Figure 3, the DMFC is preferentially activated when subjects predict events under varying levels of uncertainty based on natural sampling (Volz et al., 2003). Disregarding the level of uncertainty, the pre-SMA, BA 8, and subcortical networks including the ventral striatum and ventral tegmental area are significantly activated during prediction under uncertainty compared with prediction under certainty. Among these regions, BA 8 is the only region that shows activity changes that significantly correlates with the level of uncertainty (Volz et al., 2003). Notably, BA 8 is commonly activated regardless of whether uncertainty is caused by external or internal factors (Volz et al., 2004). Other studies also point to the activation of the frontomedian wall (typically BAs 8 and 9) using various task paradigms involving decision-making under ambiguity (Hsu et al., 2005; Yoshida and Ishii, 2006) or risk (Mohr et al., 2010; Symmonds et al., 2013). Activity in the more anterior BA 10 encodes uncertainty of inference about other people's beliefs in a strategic game (Yoshida et al., 2010).

Uncertainty is a key dimension of daily behavior that influences not only one's own decisions, but also emotions such as anxiety. The ability to tolerate uncertainty markedly differs across individuals; some people suffer from stress, discomfort, and avoidance that uncertainty induces (Mushtaq et al., 2011; Grupe and Nitschke, 2013). Affective appraisal of ambiguous faces is associated with activation in networks including the DMFC (Simmons et al., 2006). Moreover, the activation of mesial BA 8 *negatively* correlates with the degree to which subjects cannot tolerate uncertainty (“intolerance of uncertainty”) (Schienle et al., 2010). Because activation in this region increases with an increasing level of uncertainty (Volz et al., 2003, 2004), the DMFC might be necessary for coping with, or resolution of, uncertainty (Yoshida and Ishii, 2006; Schienle et al., 2010). It is possible that this function is impaired in individuals with an intolerance of uncertainty, making them unable to think or act under stressful conditions (Buhr and Dugas, 2002). A tempting hypothesis is that the avoidance of interpersonal relationships in some people with anxiety disorders may, at least in part, arise from an intolerance of uncertainty associated with inferences about others' mental states. A related question is whether individuals with a greater intolerance of uncertainty show atypical brain activation patterns during performance of ToM tasks.

It has been proposed that the neuromodulator noradrenaline may play a role in processing uncertainty (Yu and Dayan, 2005). Evidence suggests that pupil size, an indirect measure of noradrenaline levels (Aston-Jones and Cohen, 2005b), increases with increasing estimation uncertainty (Nieuwenhuis et al., 2005; Preuschoff et al., 2011; Nassar et al., 2012). Importantly, the MFC—the anterior cingulate area and adjacent frontomedian wall likely including the pre-SMA—is the major source of inputs to the locus coeruleus (Aston-Jones and Cohen, 2005a), where noradrenaline-containing neurons are abundant. Indeed, uncertainty driven by volatility modulates pre-SMA activity (Behrens et al., 2007). Another neuromodulator that may play a role in uncertainty is dopamine. It has been shown that dopamine-containing neurons in the midbrain signal uncertainty in the reward prediction (Fiorillo et al., 2003). These dopaminergic neurons preferentially project to the MFC in addition to the striatum (Williams and Goldman-Rakic, 1998). The precise contribution of neuromodulators in uncertainty processing and their impact on the subsequent coping behavior is an interesting topic of future research.

## Perception of intention

A classical definition of social psychology is that it is “an attempt to understand and explain how the thought, feeling, and behavior of individuals is influenced by the actual, imagined, or implied presence of other human beings (Allport, 1954).” The influence of the actual presence of others is indeed potent, but so is the influence of imagined or implied presence. Allport has pointed out that social influence can exist even when others are non-observable. This definition has been influential in psychology, but one might then want to ask a simple question: what is special about the definition at all in terms of social aspects of human cognition? Put in another way, what aspect best captures “social” cognition? Probably, the answer does not reside in the words “imagined or implied presence,” as one's cognition, affect, or action is also influenced by the imagined or implied presence of non-social things such as money. Instead, the answer appears to reside in the very last word “beings.” Allport's definition implicitly asks neuroscientists why people perceive a certain physical entity as a social being on one hand while viewing another entity as a non-social thing on the other. Once people “see” the mental states such as intentions in an entity, it becomes perceived as a social being and affects the way in which people think, feel, and behave. We argue that the perception of intentions in others plays a fundamental role in social cognition. The DMFC has been implicated in attention to and perception of such intentions.

Developmental studies suggest that the brain is equipped with mechanisms that make people perceive intentionality and allow for a distinction between social beings and non-social things. Infants as young as 5–8 weeks can exhibit imitative behavior in response to a person's movement at significant levels but not to the movement of artificial devices (Legerstee, 1991). Eighteen-month-old children can infer intentions from movement when it is performed by persons but not by inanimate objects (Meltzoff, 1995). They also have the ability to distinguish between intentional and accidental actions performed by others (Olineck and Poulin-Dubois, 2005). Distinguishing intentional actions from accidental actions may also be observed in non-human primates (Call and Tomasello, 1998). The sensitivity to intention in others may form the basis of human traits that people often view others' actions as caused by those others' internal dispositions (Pronin, 2008) and tend to view social agents' choices as indeterministic as opposed to viewing non-social physical events as deterministic (Nichols, 2004). Of interest is that the ability of 1-year-old infants to attend to others' intentional actions can predict the development of ToM at a preschool age (Wellman et al., 2008). Moreover, the ability of 18-month-old infants to distinguish between intentional and accidental actions is related to the development of internal state language 12 months later (Olineck and Poulin-Dubois, 2005).

The ability to perceive intentions in others may be intimately associated with the ability to direct attention to, and become aware of, one's own intention. These abilities may have similar origins in the brain. Accumulating evidence indeed suggests that at least the DMFC is concerned with both self-intention and other-intention processes.

**Figure 4:**
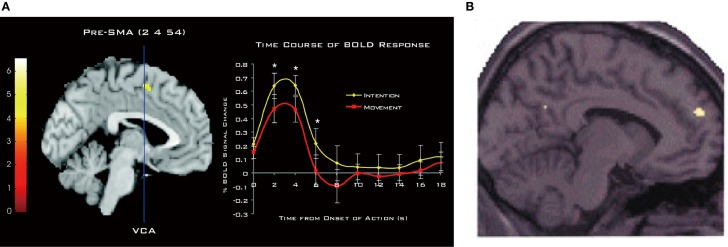
**Involvement of DMFC in attention to and perception of intention. (A)** Activation of the pre-SMA associated with attention to intention as compared to attention to movement. The time course of the hemodynamic response is shown on the right. Reprinted with permission from Lau et al. (2004). **(B)** Activation of BA 9 associated with anticipation of third-person actions as compared to non-biological agent (i.e., computer) actions. Reprinted with permission from Ramnani and Miall (2004). ^*^P < 0.005. (one-tailed paired *t*-test).

The involvement of the DMFC in intention processes was shown by Fried et al. (1991) in patients receiving electrical stimulation during neurosurgery of intractable epilepsy. They found that low-intensity stimulation in the SMA could evoke a *conscious urge* to move in a specific body part, which was often, but not always, followed by the actual movement of the same body part at high currents. A network of the MFC including the SMA, pre-SMA, and anterior cingulate cortex is strongly activated when subjects generate intentional actions that are endogenous (Libet et al., 1983; Ball et al., 1999; Yazawa et al., 2000; Cunnington et al., 2002; Fried et al., 2011), change intentional action plans (Nachev et al., 2005), or switch from automatic to intentional actions (Isoda and Hikosaka, 2007; Hikosaka and Isoda, 2010). Notably, when participants pay attention to their intention to move, rather than to their actual movement, there is an increase in activity in the pre-SMA (Figure 4A), leading the authors to conclude that pre-SMA activity reflects the representation of intention (Lau et al., 2004). Consistent with this finding, transient disruption of the pre-SMA with transcranial magnetic stimulation can reduce the temporal binding between intentional actions and their external consequences (Moore et al., 2010), which is known as an implicit measure of the sense of agency (Haggard et al., 2002). Finally, as mentioned earlier, intention to withhold an endogenously intended action activates the dorsal frontomedian cortex (BA 9).

The DMFC is also involved in the perception of intentions in others. An fMRI study showed that attributing the causation of external events to another person (other-agency) is associated with activation in the DMFC, including the SMA, caudal cingulate zone, and BA 9 (Spengler et al., 2009). Intriguingly, DMFC activity significantly correlates with individual personality traits of external action attribution (Spengler et al., 2009). As can be seen in Figure 4B, anticipating the action of intentional agents, but not that of computers, leads to the activation of a similar region in BA 9 (Ramnani and Miall, 2004). A meta-analysis of fMRI studies points to the converging activation of the pre-SMA and BA 8 in other-agency (Sperduti et al., 2011). These findings suggest that the DMFC that processes one's own intentions also processes others' intentions, supporting the view that perception of one's own intentions may, at least partly, share similar brain mechanisms to perception of others' intentions. As Frith (2002) has argued, the ToM ability requires the sense of other-agency that the actions of others are caused by their intentions. Supporting this view, the mentalizing system including the DMFC is recruited mostly when behavioral tasks describe the human agency or traits about humans, and much less so when these aspects are absent (Van Overwalle, 2011). The perception of intentions in others—be it illusory or not—is the first step in initiating many forms of interpersonal relationships. In this light, it is of importance to determine crucial factors whereby an observer perceives a target as an intentional agent (Johnson et al., 1998).

## Concluding remarks

We have reviewed the role played by the DMFC in executive inhibition, self-other distinction, prediction under uncertainty, and intention-related processing. The involvement of the DMFC in these processes may explain why the DMFC is preferentially activated when people mentalize others' internal states. We do not claim, however, that the key processes outlined above are implemented only by the DMFC. As mentioned earlier, executive inhibition also recruits the rIFG and subcortical structures (Aron et al., 2004, 2007). It seems likely that the distinction between self and others also depends on the computational operation in regions around the temporoparietal junction (TPJ) and superior temporal sulcus (STS) (Hietanen and Perrett, 1993; David et al., 2007; Farrer et al., 2008; Sperduti et al., 2011). Prediction under uncertainty can additionally recruit many regions including the dorsolateral prefrontal cortex, orbitofrontal cortex, anterior and posterior divisions of cingulate cortex, parietal cortex, lateral septal regions, pulvinar, and anterior insula (Critchley et al., 2001; McCoy and Platt, 2005; Tobler et al., 2007; Kepecs et al., 2008; Platt and Huettel, 2008; Preuschoff et al., 2008; Bossaerts, 2010; Lamm and Singer, 2010; Stern et al., 2010; Mushtaq et al., 2011; Grupe and Nitschke, 2013; Komura et al., 2013; Monosov and Hikosaka, 2013). Finally, intention processing also occurs in the inferior parietal cortex (Desmurget et al., 2009). These findings suggest that ToM is a product of global neural networks linking multiple brain regions (Frith and Frith, 2003; Gallagher and Frith, 2003; Van Overwalle and Baetens, 2009; Lombardo et al., 2010b).

Also, it is not the intention of this paper to claim that the four processes discussed are the only ones that are associated with ToM. In social life, one needs to attentively monitor the behavior of others, as it provides an important clue to understanding their mental states. The DMFC is also involved in performance monitoring in both social and non-social contexts (Ullsperger and von Cramon, 2001, 2004; Taylor et al., 2007b; de Bruijn et al., 2009; Yoshida et al., 2012). Other related processes can include simulation learning (Suzuki et al., 2012), hypothesis testing (Elliott and Dolan, 1998), and perspective-taking (Ruby and Decety, 2001, 2003) or viewpoint transformation (Wraga et al., 2005). Each of these processes activates the DMFC. Clarifying the cellular mechanisms of such higher-level cognitive processing has not been possible in non-human primates due to the complexity of tasks that monkeys can perform and, therefore, would heavily rely on experiments in humans, perhaps using a combined approach of functional imaging, single-neuron recording, and computational modeling.

The mentalizing ability allows one to infer not only the intentions of others but also their affective states. Although not reviewed in the present article, it should be mentioned that the capacity to share the feelings and emotions of others, referred to as empathy, contributes to the understanding of other people's mental states (Singer, 2006; Melloni et al., 2013). Empathy relies on limbic and paralimbic divisions of the MFC, including the anterior cingulate cortex, orbitofrontal cortex, ventromedial prefrontal cortex, as well as the anterior insula (Singer et al., 2004; Singer, 2006; Pessoa, 2008; Kennedy and Adolphs, 2012; Melloni et al., 2013). Notably, Ibanez et al. (2013a,b) have recently demonstrated that performance of emotional inference of others' feelings and thoughts can be predicted by individual differences in executive function, empathy, and a cortical potential that captures the processing of emotional stimuli, suggesting a close link between affective processing, executive function, and ToM. These findings are also in line with the proposal that emotion and cognition strongly interact in the brain and jointly contribute to behavior (Pessoa, 2008). In this regard, an important question for future research is how—in both behavioral and neural terms—the four component processes outlined here are influenced by the affective states of individuals. Future research should also investigate the mechanisms underlying interdependence between affective and cognitive processing in the context of ToM. To address these issues and understand the cellular basis of empathy, it would be useful to establish reliable markers that capture different types of emotion in non-human primates. The measurement of facial expressions combined with autonomic nervous system indexes may allow for the identification and classification of emotional states.

Social cognition, including mentalizing, is thought to be mediated by a specific set of neural circuits, often referred to as the “social brain.” Thus, an additional consideration in understanding ToM concerns how the DMFC interacts with other regions in large-scale networks. Such network perspectives are now being widely applied to the study of neurological and psychiatric disorders as well, representing a shift in emphasis from specific brain regions to specific brain networks (Menon, 2011; Castellanos and Proal, 2012; Ibanez and Manes, 2012; Kennedy and Adolphs, 2012; McCairn et al., 2013). The fact that some reports show only partial or no affection of ToM due to damage in the MFC (Bird et al., 2004; Baird et al., 2006; Shamay-Tsoory et al., 2006; Shamay-Tsoory and Aharon-Peretz, 2007) also promotes network-level considerations. Importantly, the MFC of humans and monkeys, including areas associated with mentalizing, has functional organization that shares similar patterns of coupling between each MFC subregion and the rest of the brain (Sallet et al., 2013). There is also evidence that a specific neural network covaries with the complexity of social networks in both humans and monkeys (Bickart et al., 2011; Sallet et al., 2011; Lewis et al., 2011; Kanai et al., 2012; Rushworth et al., 2013). For example, the middle part of the monkey STS has a connectivity profile that is most similar to the human TPJ (Mars et al., 2013), another crucial area in the mentalizing network. The gray matter density in the mid-STS, and that is in areas 9 and 10, increases as the complexity of macaques' social environments increase (Sallet et al., 2011). Such a temporofrontal coupling also exists even at rest, constituting the “dorsal medial prefrontal cortex subsystem” of the default mode network (Andrews-Hanna et al., 2010). Furthermore, the DMFC is increasingly recruited in the default mode network as the social complexity increases (Mars et al., 2012). These findings may suggest that the STS and the DMFC are integrative “hubs” in large-scale social brain networks for predicting others' intentions and behavior. Activity in these hubs, and interactions between them, may be occurring more frequently when animals are in larger social groups, because they have to make and adjust more predictions about what other members will do in a given context. This conjecture is supported by activity in DMFC that reflects expectations about what another agent will do and errors in such predictions (van Schie et al., 2004; Suzuki et al., 2012; Yoshida et al., 2012) and is also in line with the proposal that the frontotemporal network plays a key role in context-driven predictions (Bar, 2004, 2009; Barrett and Bar, 2009) particularly under social situations (Ibanez and Manes, 2012). It should be emphasized that social cognition processes, including the prediction of others' intention and behavior, are embedded in specific contextual circumstances.

The monkey STS contains many neurons that are selective for the direction of the face (or head), eye gaze, and body of another agent rather than for its identity (Perrett et al., 1985, 1992; Wachsmuth et al., 1994; De Souza et al., 2005), suggesting that this cortical area is important in determining where the target agent is attending. Moreover, parts of the STS contain neurons that are sensitive to other sources of social information, such as motion of others' body parts (Hietanen and Perrett, 1993; Oram and Perrett, 1994). Furthermore, the activity of those neurons is likely to be modulated by the intentionality of another's actions (Jellema et al., 2000). Thus, the monkey STS, identified as most similar to human TPJ, may be involved in detecting whether the target is animate or not and understanding what the target's intention is, at least in a rudimentary form. Such signals may then be conveyed to the DMFC (Seltzer and Pandya, 1989; Luppino et al., 2001), where the information is integrated with contextual information, predictions are made about what the agent is going to do, and appropriate behavior is organized to meet a contextual need as well as one's own goal. Perhaps, during social interactions, the four processes are simultaneously engaged in the network to predict others' intention. The challenge for future research is to determine the biological underpinnings and computational formulations of such concurrent network operations.

It appears that the region activated in mentalizing tasks is often more anterior, albeit with some overlap, than the regions typically activated in some of the component processes outlined in the present article, such as executive inhibition, prediction under uncertainty, and attention to or perception of intention. Whereas such a regional differentiation may suggest that the anterior DMFC plays a role in integrating different component processes to support the appropriate mentalizing operation in a task at hand, it may also support the existence of another function that is crucial for recruiting the more anterior part. One plausible hypothesis is that the degree of recursive inferences or simulations involved in mentalizing determines the degree of activity in this region. Adaptive success in social life, in particular when competing against an intelligent adversary, requires iterated steps of reasoning about each other's mental states, for example, “what you think the others think about what you think.” It is such a process of higher-order recursions that preferentially recruits the anterior DMFC (BA 10) (Hampton et al., 2008; Coricelli and Nagel, 2009). Another hypothesis that could account for the functional gradient between the more caudal vs. rostral DMFC is that the former is associated with a general role in perceiving intentions in others and the latter plays a specific role in inferring the *content* of others' intentions. This intriguing hypothesis is testable using neuroimaging techniques with human subjects.

People do not mentalize an object such as a car or computer as long as they do not assume the mental states in it. It is the subjective perception of a mind in the target that triggers mentalizing and social interactions. The condition in which the DMFC becomes active is not confined to inferences about other human beings, but can also include those about non-human animals (e.g., dogs) (Mitchell et al., 2005), which are generally believed to have mental states. Notably, even early infants have biological mechanisms that make them sensitive to animacy and intentionality. Perceiving the mental states such as intentions in others makes the world around us *social* and therefore underlies virtually all kinds of social interactions. Neuroscientists are given the great opportunity to challenge the following profound questions: “What neural mechanisms make observers interpret that a certain physical entity has a mind?” and “what neural mechanisms underlie the perception of intentionality in others' actions?” Of course, these questions are inevitably related to the problem of free will.

### Conflict of interest statement

The authors declare that the research was conducted in the absence of any commercial or financial relationships that could be construed as a potential conflict of interest.
